# Tea Plant (*Camellia sinensis*): A Current Update on Use in Diabetes, Obesity, and Cardiovascular Disease

**DOI:** 10.3390/nu15010037

**Published:** 2022-12-21

**Authors:** James Michael Brimson, Mani Iyer Prasanth, Kishoree Krishna Kumaree, Premrutai Thitilertdecha, Dicson Sheeja Malar, Tewin Tencomnao, Anchalee Prasansuklab

**Affiliations:** 1 Natural Products for Neuroprotection and Anti-Ageing Research Unit, Chulalongkorn University, Bangkok 10330, Thailand; 2 Department of Clinical Chemistry, Faculty of Allied Health Sciences, Chulalongkorn University, Bangkok 10330, Thailand; 3 Siriraj Research Group in Immunobiology and Therapeutic Sciences, Faculty of Medicine Siriraj Hospital, Mahidol University, Bangkok 10700, Thailand; 4 College of Public Health Sciences, Chulalongkorn University, Bangkok 10330, Thailand

**Keywords:** *Camellia sinensis*, diabetes, cardiovascular disease, obesity, metabolic syndrome, natural products, EGCG, catechin, therapeutic mechanisms

## Abstract

The tea plant (*C. sinensis*) has traditionally been consumed worldwide as “tea” for its many health benefits, with the potential for the prevention and therapy of various conditions. Regardless of its long history, the use of tea plants in modern times seems not to have changed much, as the beverage remains the most popular form. This review aimed to compile scientific information about the role and action of tea plants, as well as their status concerning clinical applications, based on the currently available evidence, with a focus on metabolic syndrome, mainly covering obesity, diabetes, and cardiovascular disease. It has been recognized that these diseases pose a significant threat to public health, and the development of effective treatment and prevention strategies is necessary but still challenging. In this article, the potential benefits of tea plants and their derived bioactive components (such as epigallocatechin-3-gallate) as anti-obesity, anti-diabetic, and anti-cardiovascular agents are clearly shown and emphasized, along with their mechanisms of action. However, according to the status of the clinical translation of tea plants, particularly in drug development, more substantial efforts in well-designed, randomized, controlled trials are required to expand their applications in treating the three major metabolic disorders and avoiding the toxicity caused by overconsumption.

## 1. Introduction

### 1.1. Tea Plant (Camellia sinensis) and Its Characteristics

*Camellia sinensis* is a flowering plant from the family Theaceae, the leaves of which are harvested to produce tea. In most of its modern forms (white tea, yellow tea, green tea, oolong, and dark tea, including Pu-erh tea and black tea), it is harvested from one of two varieties of *Camellia sinensis*: *C. sinensis* var. sinensis and *C. s.* var. assamica. The plant is commonly known as the tea plant, tea shrub, or tea tree (not to be confused with Melaleuca alternifolia, the source of tea tree oil, or Leptospermum scoparium, the New Zealand tea tree). The tea plant is native to East Asia, possibly originating in southern China, bordering Myanmar and India [1,2,3]. The Chinese small-leaf variety (*C. s.* sinensis) and the large-leaved Assamese plant (*C. s.* assamica) are the two varieties of tea plants used to produce tea. The tea plant will grow into a tree if allowed, but it is usually cultivated and trimmed to waist high to make harvesting the leaves easier.

The tea plant leaves vary in size depending on the variety, ranging from 4 to 15 cm long and 2 to 5 cm wide. The leaf buds or tips are picked to produce tea, and the age of the leaf affects the quality of the tea made as the chemical compounds change as the leaf ages. The caffeine content of a tea leaf is approximately 4%, with other phenolic compounds, including the catechins (such as epigallocatechin-3-*O*-gallate (EGCG)), theaflavins (such as TF-1), tannins, and flavonoids, making up the majority of the other constituent compounds.

### 1.2. Traditional and Modern Usage

#### 1.2.1. Traditional Usage

**Figure 1:**
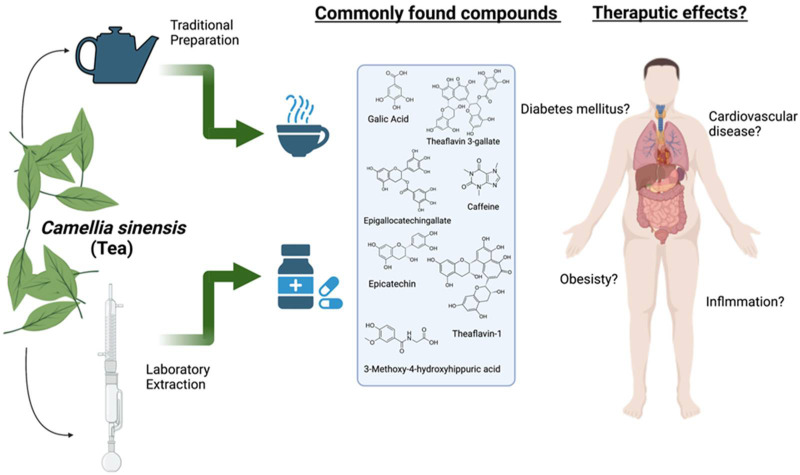
The extraction of chemicals from tea leaves in the laboratory or in the teapot, the therapeutic compounds that are found, and the diseases they may benefit. (Created with BioRender.com).

The leaves of *C. sinensis* are traditionally prepared as a concoction, mainly tea, one of the most globally consumed beverages. The finished tea leaves of *C. sinensis* can be varied into six types: white, green, oolong, black, dark, and yellow tea, according to the quantification of their major components and fermentation process [4]. The history of drinking tea is deeply rooted in several countries in Southeast Asia, which has been the tea plant’s origin for nearly fifty centuries [5]. The tea plant was used for the pleasant fragrance of tea leaves and was later recognized as a medicinal herb, starting from the Tang and Song Dynasties [6]. Black tea is predominantly consumed and favored worldwide in Eastern and Western countries. At the same time, green tea is preferable in Asian countries (including China, Japan, and India) [7] and is extensively used in the traditional Chinese medicinal system [6]. The therapeutic uses of green tea to eliminate thirst, heat, and phlegm and to act as a diuretic and an anti-inflammatory were officially recorded in classical literature. White tea is also prescribed to treat measles, flu, and diabetes in Chinese folklore [8]. Furthermore, the intake of tea, in general, is traditionally recommended for a broad variety of ailments, including headaches, body aches, pains, indigestion, and depression, as well as for detoxification and as an energizer to prolong life (Figure 1) [9].

#### 1.2.2. Modern Usage

In addition to brewing *C. sinensis* leaves with hot water as a tea, the leaves (i.e., originally black and green tea) were later introduced to be the primary substrate for tea fermentation in a recent popular drink called kombucha [10], which has been reported as having health benefits due to its antioxidant, anti-microbial, anti-diabetic, and anti-carcinogenic properties [11]. The green tea leaf extract is also used as a dietary supplement for weight control [12,13], while the extracts of green, black, and white teas have grown in interest as skincare products [14]. The activities related to health promotion, disease prevention, and treatment are contributed to by the active compounds in the tea plant, including polyphenols, flavanols, catechins, catechin gallates, adenine, caffeine, theobromine, theophylline, gallic acids, tannins, and gallotannin [15,16]. Anti-obesity is reported explicitly as the effect of the theaflavins in black tea [17] and the polysaccharides in green tea [18]. The anti-obesity efficacy is even more remarkable when using polysaccharides in combination with polyphenols [18]. For anti-diabetes, the responsible active compounds, particularly in black tea, are still ambiguous as the evidence of this activity is found in the tea extracts, which are rich in either theaflavins and thearubigins (from the black tea aqueous extract) or catechins (from the green tea aqueous extract), instead of the purified compounds themselves [19]. Only one study suggests that EGCG isolated from the green tea leaves with 94% purification enhanced glucose tolerance in diabetic rodents [20]. The cardiovascular protective effect of black and green tea has also been widely studied, and it has been demonstrated that this protective property results from flavonoids in the tea leaves [21]. Consuming a cup of black tea with 237 g in 236.6 mL (~280 mg total flavonoids) or a cup of green tea with 245 g in 236.6 mL (~338 mg total flavonoids) daily was able to lower the risks of cardiovascular events [21]. EGCG is reported to be a tentative compound contributing to this property via the protection of endothelial function [22], whereas catechins and epigallocatechin (EGC) acted through anti-platelet activity [23,24]. More interestingly, *C. sinensis* leaves may even possess anti-SARS-CoV-2 (severe acute respiratory syndrome coronavirus 2) properties, which could be the effect of the catechins therein [25]; so, drinking tea or taking the extracts could potentially help as a prevention against this novel coronavirus infection and could reduce the viral progression in the mouth and the upper gastrointestinal and respiratory tracts [26].

## 2. Role of Tea Plant and Related Compounds in Diabetes Mellitus

Diabetes mellitus is a group of physiological dysfunctions characterized by a hypoglycemic state, insulin deficiency, inadequate insulin secretion, or excessive glucagon secretion [27]. The World Health Organization (WHO), along with the American Diabetes Association (ADA), identified four types of diabetes mellitus: type 1 diabetes mellitus (T1DM), type 2 diabetes mellitus (T2DM), “other specific types”, and gestational diabetes [28,29], wherein T1DM and T2DM are the prominent ones, and among them, the latter is predominant. In simple terms, T1DM primarily involves autoimmune insulin deficiency, whereas T2DM is contributed to by the pathological state of insulin deficiency and insulin resistance [27].

**Figure 2:**
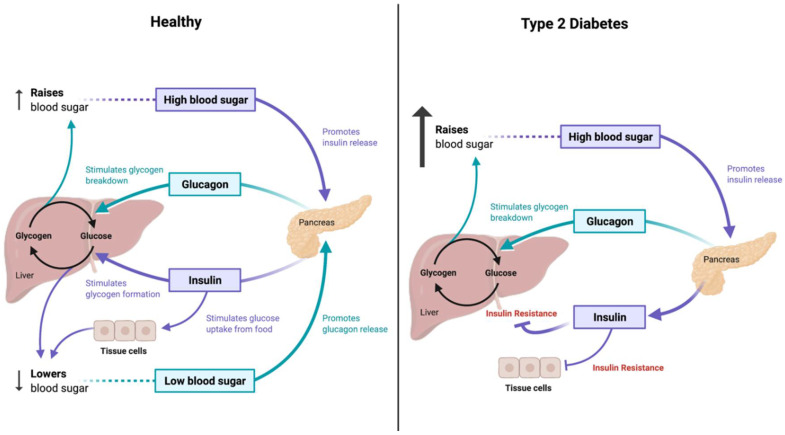
Glucose metabolism in type 2 diabetes. (Created with BioRender.com).

Diabetes accounts for altered insulin secretion and resistance, along with the decreased sensitivity of target organs towards insulin, which is eventually followed by fat, protein, water, electrolytes, and other metabolic disorders (Figure 2) [27]. An imbalance in the gut microbiome can also have a role in determining diabetes as it helps to break down carbohydrates, which could eventually lead to diabetes [30]. The metabolites produced by the microbiome, such as short-chain fatty acids (SCFAs), amino acid derivatives, and secondary bile acids, can participate in metabolic and immune processes, which, when altered, could lead to the development of diabetes [31].

Continuous glucose monitoring (CGM) is a powerful tool in the management of diabetes that has helped patients with diabetes to achieve better glycemic control as compared to the traditional systems. It helps to reduce the frequency and severity of hypoglycemia [32]. The management of diabetes in clinical practice has many challenges: quickly interpreting a large volume of self-monitored blood glucose data, ensuring safe and accurate titration of basal insulin, managing patients on insulin pump therapy, and synthesizing glycemic data into actionable reports to improve patient outcomes. Technological advancements are emerging to solve some of these challenges [33].

Presently, a variety of natural compounds, including flavonoids, glycosides, terpenoids, and alkaloids, which include epigallocatechin-3-gallate, curcumin, allicin, quercetin, genistein, capsaicin, rhein, pectin, berberine, and resveratrol, among many others, can be used in the treatment of diabetes [34,35,36]. These compounds majorly target oxidative stress, endoplasmic reticulum stress, and metabolic and inflammatory responses in the host [37]. Additionally, the intestinal flora, known to regulate several T2DM pathway steps, can also mediate diabetes along with natural compounds [27].

### 2.1. Tea Plant’s Anti-Diabetic Roles

Green tea phenolic constituents stimulated pancreatic beta cells to increase postprandial insulin, consequently improving the activity of the pancreas [38]. EGCG can inhibit the proliferation and differentiation of adipocytes, thereby increasing glucose reception by the cells through protein kinase by AMP activation [39]. Another study was conducted in rats on glucose tolerance and insulin sensitivity after green tea supplementation, which showed lower concentrations of fasting glucose, insulin, and triglycerides [40,41].

EGCG was reported to improve the parameters of diabetic mice and to increase the Firmicutes/Bacteroidetes ratio and that of the Lactobacillus species [42]. At the family level, EGCG increased Christensenellaceae and decreased the Enterobacteriaceae and Proteobacteria proportions [39]. Moreover, EGCG changed the gut microbiota composition of patients with obesity by regulating the bile acid signaling pathway [43].

A meta-analysis of 17 randomized controlled trials with both healthy subjects and patients with chronic diseases such as obesity, T2DM, or hypertension showed that green tea consumption significantly reduced the circulating levels of fasting glucose, HbA1c, and fasting insulin [44]. In another meta-analysis study, subjects who consumed four or more cups of tea per day had a 20% lower risk of T2DM [45].

Even though there are not many direct studies on the effect of green tea in mediating diabetes clinically, a few studies have been conducted on diabetic subjects with green tea supplementation and have characterized various parameters. A meta-analysis study indicated that green tea consumption significantly decreased body weight, body mass index, and body fat in T2DM patients in long-term interventions of more than 8 weeks, at lower doses of green tea, in overweight patients [46]. Additionally, green tea supplementation improved lipid profile by reducing the serum concentrations of triglycerides in patients with T2DM and by reducing the serum concentrations of triglycerides and total cholesterol [47]. Green tea could also significantly reduce the circulating levels of C-reactive protein, without affecting the malondialdehyde and total antioxidant capacity in T2DM patients [48]. Adiponectin, the key component in the interrelationship between adiposity, insulin resistance, and inflammation [49], is inversely proportional to the incidence of diabetes in different populations [50]. In a meta-analysis, supplementing green tea was reported to increase the adiponectin concentrations in patients with T2DM, thereby reducing the possibility of diabetes [51].

Green tea catechins have been shown to actively modulate the activity or expression of several receptors and enzymes involved in the absorption, metabolism transport, and synthesis of carbohydrates [52]. Digestive enzymes are inhibited in vitro by green tea catechins, including alpha-amylase, intestinal sucrase, alpha-glucosidase, and gastric H^+^, K^+^-ATPase [53,54,55,56].

### 2.2. Clinical Applications of Tea Plant for Treatment of Diabetes

A recently reported systematic review and meta-analysis of the randomized controlled trials on the effect of the supplementary intake of green tea on fasting plasma glucose, fasting insulin, hemoglobin A1c, and the Homeostatic Model Assessment for Insulin Resistance (HOMA-IR) in patients with T2DM selected only 14 articles out of 780, after excluding animal studies, unrelated studies, or those with insufficient data. With this, it was concluded that the supplementary intake of green tea had no significant effect on fasting plasma glucose, fasting insulin, HbA1c, and HOMA-IR in patients with T2DM [57], indicating the need for more randomized controlled trials to establish the role of green tea in extending anti-diabetic effects.

Green tea and its constituents have been reported to positively improve several physiological parameters in clinical subjects with diabetes, such as body weight, body mass index, body fat, and lipid profile, thereby improving living conditions. Even though green tea is marketed as one of the major constituents in the prevention of and reduction in diabetes, more clinical studies are required to substantiate these claims and define a suitable dose range. However, green tea could potentially improve the physical conditions of diabetic patients according to our current knowledge. However, the dose required for controlling diabetes still needs further study.

## 3. Role of Tea Plant and Related Compounds in Obesity

Obesity is a serious health concern and, sadly, one of the most prevalent conditions among most of the populations of the world. Obesity, as defined by the World Health Organization (WHO) 2021, is one abnormality by which the body stores excessive fat that eventually alters the standard metabolic mechanism for a healthy body [58]. According to data from the WHO, a body mass index (BMI) ≥ 25 kg/m^2^ is described as overweight, whereas a BMI ≥ 30 kg/m^2^ is specified as obesity for adults. The imbalance of energy utilization and consumption directs the body to the storage of excess energy as triglycerides (TGs) in the adipocytes, leading to enhanced adipogenesis, known as obesity. 

**Figure 3:**
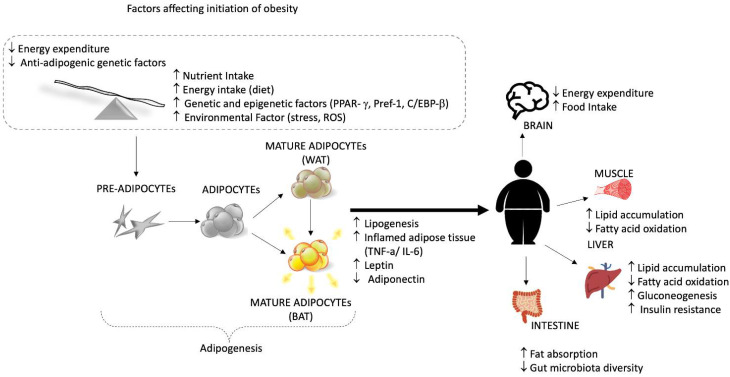
Schematic representation of factors influencing the onset of obesity and adipogenesis and the adverse effect of being overweight on the body.

Currently, obesity is a complex form of the condition as its onset is associated with several other diseases, such as diabetes, cardiovascular disease, and cancer [59,60,61,62]. Adipose is a form of the active endocrine system, secreting various bioactive molecules called adipokines. The proper functioning of adipokines reflects healthy adipose tissue. In contrast, its dysfunction may link to the adverse outcomes of obesity, including insulin resistance, type 2 diabetes, fatty liver disease, and chronic inflammation (TNF-α, IL-6-8-10, transformation growth factors β, interferon-γ, C-reactive protein/CRP, and fatty acid-binding protein4/FABP4) [60,63,64,65]. The pathogenesis of obesity involves the dysfunction of multiple factors within the system, such as different hormones, the neural signal from the brain, the stomach, the adipose tissues, and the pancreas (Figure 3) [66]. The varying signals from these organs regulate the intake and actual requirement of food or the energy source [67].

The development of anti-obesity drugs helps to target these signals within the body, including inhibitors for TGs, appetite suppressants, lipase inhibitors, and others. Controlling diet intake and physical activity is widely accepted as an effective treatment for obesity, but it might be challenging to achieve. The FDA has approved several anti-obesity drugs, such as phentermine and orlistat, but such drugs have side effects [68,69]. While most of the drugs are mechanistically designed to act as anti-obesity drugs, they fail to perform efficiently, and they raise concerns about side effects during long-term treatments. For example, appetite suppressants can affect the central nervous system (CNS), causing CNS-related side effects such as anxiety, depression, and insomnia [70,71,72]. 

With the concern over the pharmacological drugs, the implications of the bioactive components from natural resources to mitigate obesity with lesser side effects are widely investigated. Several earlier studies have proven the beneficial impact of tea consumption on obesity. For example, an epidemiological study conducted with approximately six thousand participants from 2003 to 2006 showed that hot tea consumption was proportional to obesity [73]. This section highlights the recent updates regarding the bioactive components of tea and their mechanisms of action in controlling obesity and discusses the underlying mechanisms of action.

### 3.1. Anti-Obesity Mechanisms of Tea Plant

#### 3.1.1. Action on Digestive Enzyme Inhibition

The inhibition of these enzymes and bioactive components in tea can lower the absorption rate of sugars and fats by decreasing weight gain. Pancreatic lipase inhibitor is one of the main beneficial factors of hydrolyzed lipids (fats, oils, and TGs); thus, inhibiting the lipase could be a major way to prevent weight gain. Studies have shown that the polyphenolic contents in tea can inhibit fat digestion by inhibiting pancreatic lipase (PL). It was discovered that EGCG prevented PL activity in vitro (IC50 = 7.5 µmol/L) with regard to the substrate concentration. Similar results were also shown by the polyphenolic contents from the black tea [74]. Another in vitro study reported that the consumption of green tea and catechins could successfully reduce the activity of PL, which might eventually help in weight reduction [75].

Meanwhile, studies have also supported the reduction in these enzymes by reducing their gene expression. Green tea extract has been shown to downregulate the mRNA expression of the genes responsible for hepatic lipid uptake [76]. The polyphenolic content of tea prevents the digestion of fat and carbohydrates by inhibiting enzymes, including digestive enzymes such as glucosidase and amylases. In a study on oolong tea polyphenols, EGCG and EGCG”Me had a significant inhibitory effect against a-amylase and its IC_50_ values [77]. In research by Takashi et al., the extraction of black tea showed that black tea extract affects carbohydrate absorption by modulating postprandial hyperglycemia. They confirmed the inhibitory effect of black tea on the degradation of the disaccharides at both the in vitro and the in vivo levels [78]. C57BL/6J mice fed with a high-fat diet (HFD) were supplemented with green and black tea for 14 weeks, and they reported that the suppression of the high-fat-diet evoked hyperglycemia by stimulating the expression of glucose transporter-4 [79]. In another study using *Caenorhabditis elegans* as a model to explore the water extract of Pu-erh tea on reducing lipid synthesis, the study reported the downregulation of the genes responsible for the fat regulator SBP-1 (SREBP) and its target stearoyl-CoA desaturase (SCD), a key enzyme in fat synthesis [80].

#### 3.1.2. Action on Intestinal Microbiota

Growing evidence suggests that tea polyphenols have an indirect effect on obesity via modulation of the intestinal microbiota and thus acting as a prebiotic supplement. Several animal trials have reported the significant impact of tea extracts (green tea, black tea, fermented green tea, oolong tea, and Fuzhuan brick tea) on the improvement of healthier gut microbiota, eventually leading to the reduction in body weight in the mice fed a high-fat diet [81,82,83,84,85]. The beneficial effect was a result of the inducing of the number of beneficial bacteria, which may be helping in regulating energy metabolism within the body. Moreover, Pu-erh tea also significantly impacted weight loss in the mice fed a high-fat diet by interfering with the fat accumulation and adipose inflammation by modulating the microbiota [86]. A study reported an increased number of bacteria responsible for producing short-chain fatty acids (SCFA) after green tea was received every day for the following two weeks [87]. Another clinical study reported an increase in probiotic *Bifidobacteria* when the subjects substituted their daily water intake with green tea for the next ten days [88]. Tea consumption has been linked to the reversal of the host body changes due to obesity and to the increase in overall microbial diversity [89].

#### 3.1.3. Action on Energy Regulation

There are two types of adipose tissue, namely white adipose tissue (WAT) and brown adipose tissue (BAT) [90]. WAT stores energy by retaining TGs and is also responsible for hormonal secretion, inflammation, and insulin resistance, whereas BAT is responsible for thermogenesis, which regulates the body temperature [91]. Several drugs are meant to target BAT to treat obesity. It was shown that feeding with an extract from green tea could prevent weight gain by increasing the thermogenesis in BAT by activating the b-adrenoceptor in rats fed with a high-fat diet (HFD) [92]. Tea consumption can increase energy expenditure to disintegrate the excessive stored energy. Such a process can be achieved by activating BAT, the induction of the browning of WAT, and the upregulation of uncoupling protein-1 (UCP-1) through the AMPK signaling pathway and the managing of the energy homeostasis, which is a crucial process in maintaining body weight [93,94]. For instance, tea extracts such as from black tea, oolong tea, and Pu-erh were reported to induce the browning of WAT and to suppress adiposity activity via the overexpression of UCP-1 and the activation of AMPK [95].

#### 3.1.4. Action on Adipose Tissue

Changes in the size and number of adipocytes and the differentiation of adipocytes can also trigger the onset of obesity. The consumption of tea has been associated with the reduction in the number, size, and differentiation of adipocytes, providing an anti-obesity impact. The previous study reported that the treatment with oolong tea has a beneficial effect on the reduction in the size of adipocytes, the body weight, the lipid content in the liver, and the serum in obese mice fed with a high-fat diet [96]. Adiponectin is a cytokine exclusively secreted by adipose tissues, and the lower level of adiponectin is linked with the pathogenesis of obesity. At the genetic level, the expression of adiponectin is mainly structured by a nuclear transcription factor named peroxisome proliferator-activated receptor (PPAR-γ) [97]. A study on rats fed with a high-fat diet and green tea extract (GTE) illustrated that GTE consumption led to the upregulation of adiponectin mRNA expression, possibly by inhibiting the ERK-1/2 signaling pathway by alleviating PPAR-γ phosphorylation [98].

Similarly, another study showed that green tea and black tea treatment could suppress adipocyte differentiation and the genes responsible for adipocyte differentiation (PPAR-γ, Pref-1, C/EBP-β) in the perirenal fats of obese rats [99]. Another mode of targeting obesity through tea consumption is the reduction in nutrient digestion and absorption. It has been reported that tea consumption can interfere with nutrient digestion, leading to a lower energy intake. The enterocytes absorb the dietary lipid during digestion in the intestine; so, targeting the lipid update protein during digestion could be one of the processes in treating obesity with tea. It has been shown that consumption of EGCG has helped to reduce body weight and increase the lipid content in the fecal output in +mice fed with HFD [100].

### 3.2. Clinical Applications of Tea Plant for Treatment of Obesity

The major bioactive components in the different teas are EGCG in green tea, EGCG’’Me in oolong tea, and theaflavins in black tea, as well as other polyphenols, which have an anti-obesity impact. Green tea consumption has been widely studied for its anti-obesity effect due to its higher bioavailability and more potent antioxidant properties. Recent studies have proven that that all types of tea possess the anti-obesity effect, irrespective of their fermentation process. Table 1 summarizes the previous reports on the modulatory effects of tea plants against obesity in human clinical studies. However, further clinical research is still required, including research on black tea and oolong tea, to understand better their anti-obesity effects. At the same time, the higher consumption of green tea has been associated with liver dysfunction. In the future, it is essential to explore further the doses and duration of tea consumption that could have a healthier impact and lesser adverse effects.

## 4. Role of Tea Plant and Related Compounds in Cardiovascular Disease

**Figure 4:**
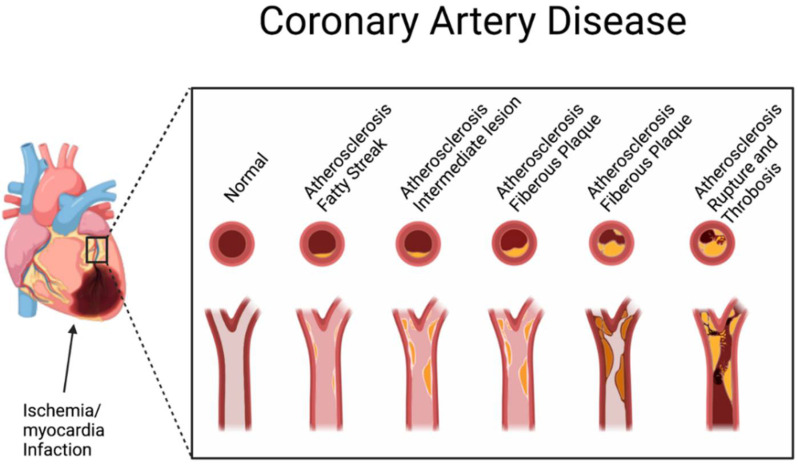
The stages of coronary artery disease development. (Created with BioRender.com).

Cardiovascular disease (CVD) is the leading cause of death globally, accounting for approximately 32% of all deaths (WHO 2021), and in the US, it is estimated that one in every three deaths is attributable to CVD [112,113]. It is the condition in which the blood vessels in the body become accumulated with plaques which disrupt the normal blood flow in the host leading to a series of damaging results, including abnormal heart rhythms, complications in the heart valve, heart failure, heart attack, and stroke (Figure 4) [112,114,115,116]. Many different conditions, such as coronary heart disease (CHD), cerebrovascular disease, peripheral vascular disease, rheumatic heart disease, congenital heart disease, deep vein thrombosis, and pulmonary embolism, can be developed due to CVD [117]. Other metabolic disorders, such as diabetes (explained in the previous section) and oxidative stress, could also lead to CVD. The excessive production of reactive oxygen species (ROS) induces cellular abnormality within the vasculature that may eventually contribute to cardiac dysfunction [118]. Obstructive sleep apnea or sleep-disordered breathing also poses a significant risk factor for major cardiovascular diseases, including arterial hypertension, ischemic heart disease, heart failure, rhythm/conduction disturbances, and cerebral stroke [119,120].

Unhealthy lifestyle habits, including a poor and unbalanced diet, lack of physical exercise, obesity, excessive smoking, and drinking, can lead to CVD. Risk prediction models can be used to understand and manage CVD and have become an essential part of the clinical decision making [121]. Many risk prediction models for CVD use one data point per patient (usually at the baseline), such as the widely used Framingham risk score, which predicts the risk of coronary heart disease [122], or QRISK3, which predicts risk of CVD in a subset of the UK population and is widely used in CVD risk stratification in the UK [123].

Multitargeted natural agents have gained the utmost attention as novel drug candidates in the emerging pharmaceutical sciences era in combating CVD and other diseases [124,125]. The natural products also provide safety, multitargeting properties, and affordability [126,127]. Various nutraceuticals have been reported to act against CVD, such as cardamonin [126] and Hydroxysafflor yellow A [128], among many others. The role of *C. sinesis* against CVD is explained in detail in the following section.

### 4.1. Anti-Cardiovascular Disease Mechanisms of Tea Plant

The role of tea and its flavonoids in preventing and reducing the risk of CVD have recently been described in detail [129,130]. Tea polyphenols can reduce systolic and diastolic blood pressure, with mean effects ranging from −1.94 to −2.08 mmHg for systolic and −1.71 to −1.94 mmHg for diastolic BP, respectively [131]. Daily tea intake as part of a healthy habitual dietary pattern may be associated with lower risks of CVD and all-cause mortality among adults. A linear meta-regression study showed that each cup (236.6 mL) in the daily tea consumption (estimated 280 mg and 338 mg total flavonoids/d for black and green tea, respectively) was associated with an average 4% lower risk of CVD mortality, a 2% lower risk of CVD events, a 4% lower risk of stroke, and a 1.5% lower risk of all-cause mortality [21]. A separate meta-analysis showed that increasing tea consumption by three cups per day significantly reduced the risk of both CVD and mortality by other causes [132].

In a cohort study, the researchers found that the consumption of green tea reduced the possibility of mortality caused by CVD. A cross-sectional observation in elderly women has shown that large amounts of tea consumption and excretion of high 4-O-MGA help to lower blood pressure by aiding in endothelium-dependent vasodilation and modulating the level of nitric oxide in the system [129]. Quercetin and the associated flavonoids in tea were reported to reduce blood pressure, oxidative status, and damage in animal models of hypertension [133,134]. L-theanine, another component, reduced blood pressure in hypertensive rats [134].

The regular consumption of tea and its active ingredients, such as epigallocatechin gallate, may be beneficial in reducing the markers of oxidative stress and inflammation, apart from enhancing nitric oxide bioavailability and lowering blood pressure. Moreover, reducing oxidized low-density lipoprotein and C-reactive protein levels could be a sign of improved endothelial function in individuals at increased risk of developing CVD [135]. Theaflavins and EGCG could reduce oxidative stress markers such as malondialdehyde and dityrosine levels. They could also induce epithelial nitric oxide synthase to produce NO and vascular relaxation [136].

Black tea, with its antioxidants such as flavonoids, could lower the risk of heart disease by preventing the oxidation of LDL cholesterol and preventing damage in both the bloodstream and at the artery walls. Additionally, these flavonoids can aid in improving cardiac muscle function and coronary vasodilation, along with reducing the formation of clots, thereby protecting the heart from CVD. Studies have shown that as few as three cups of tea per day can improve heart health [137]. Epidemiological studies suggested that the consumption of black tea decreased plasma uric acid, C-reactive protein, glucose, triglyceride, the LDL/HDL ratio, and plasma cholesterol levels. Another study reported that black tea consumption increased the blood flow in the brachial artery of patients with CVD [136].

The effect of tea and its polyphenols in delivering protection from CVD can be attributed to the effects of tea flavonoids on endothelial function, nitric oxide-dependent vasorelaxation, and blood pressure and on improving blood pressure flow by enhancing endothelial nitric oxide bioavailability [138].

### 4.2. Clinical Applications of Tea Plant in Cardiovascular Disease

The daily ingestion of green tea containing 200–300 mg of EGCG has a good effect on cardiovascular and metabolic health [129]. An umbrella review was performed on the effect of tea consumption on CVD diagnosis or incidence, stroke events, blood pressure, endothelial function, blood lipids and triglycerides, inflammatory markers, and mortality, based on the systematic reviews published in the last decade. The study reported that consuming two cups of unsweet tea per day consistently delivers the correct levels of flavonoids to potentially decrease CVD risk and its progression [138].

However, it should also be noted that the consumption of tea has some harmful effects as well. The primary side effects of tea documented in human studies are hepatotoxicity and gastrointestinal disturbances (i.e., vomiting and diarrhea) after a high-dose supplemental intake [138]. In a particular study, those in the highest tertile of tea and caffeine consumption had an elevated risk of CVD, which the authors postulate to be because of the high consumption of black tea when compared to green tea, as well as the addition of sugar to tea [139]. The effects of tea consumption on health are expected to be modulated by the quantity and quality of consumed tea [138]. For instance, the addition of sweetener, milk, or other additives and the variations in the level of polyphenols could play a massive role in the health benefits imparted by tea. Additional clinical research is therefore essential to completely elucidate the effects of tea flavonoids on the markers of CVD, which could bring more clarity.

## 5. Conclusions and Perspectives

Obesity, diabetes, and cardiovascular disease, collectively called metabolic syndrome, have been recognized as significant threats to public health. The development of effective treatment and prevention strategies is necessary but still challenging. Tea made from the tea plant (*C. sinensis*) leaves is one of the most consumed beverages worldwide. It is rich in natural compounds that are believed to provide multiple benefits for our health, including the common metabolic syndrome. Since ancient times, the tea plant in the form of tea has been used for the prevention and treatment of a variety of disease conditions, and the use of this plant seems not to have changed much as the beverage remains the most popular form. Several types of tea from this plant have been developed through variations in harvesting and processing methods, leading to differences in the quantity and quality of the active compounds consumed and the subsequent effects produced. In this review, several pre-clinical and clinical studies revealed that the primary form of tea with significant reports of benefits towards the three major metabolic diseases is green tea, followed by oolong tea, black tea, and Pu-erh tea. Among the various chemical compounds found in tea plants, a catechin EGCG seems to be the significant component responsible for the corresponding bioactivities. On the other hand, while a substantial amount of pre-clinical research on the potential benefits of tea plants and their derived bioactive components has been conducted, there was inconclusive evidence on their effects in clinical studies. In addition to that, it has not been approved as a treatment for any specific indication until now. Several products of tea plant extracts are being sold on the market as capsule dietary supplements with broad but unproven efficacy claims. According to the status of the clinical applications of tea plants, it is highly encouraged to put more substantial efforts into well-designed, randomized, controlled trials to achieve the maximum benefits of this plant for actual clinical use.

## Figures and Tables

**Table 1:** Modulatory effects of tea plant against obesity in human clinical studies.

Treatment	Dose of Tea	The Objective of the Study	Initial BMI of Subjects	Number of Participants	The Outcome of the Study	Ref
High dose green tea extract	856.8 mg of EGCG for 12 weeks	lipid profile and obesity-related hormone	>27 kg/m^2^	115 women	Body weight ↓ BMI ↓ Total cholesterol ↓ LDL plasma levels ↓ Adiponectin↑	[12]
Green tea along with a-glucosyl hesperidin	146mg EGCG for 12 weeks	Anti-obesity effect	≥25 kg/m^2^	60 healthy Japanese males and females aged between 30–75 years	Bodyweight ↓ Triglycerides ↓ Body fat percentage ↓ Visceral fat ↓ BMI ↓ LDL/HDL ratio ↓	[101]
Minnesota decaffeinated green tea	843 mg of EGCG for 12 months	Obesity-associated hormones, glucose homeostasis	≥25 kg/m^2^	937 postmenopausal women	Non-significant relation of decaffeinated green tea with a reduction in body weight and BMI and no alteration in mean hormone concentration or energy intake.	[102]
Green tea as a dietary supplement in Thais	250 mg capsule of green tea capsules from Herbal One Co., Ltd. for 12 weeks	Effect on weight reduction and associated metabolic risks	>25 kg/m^2^	60 overweight subjects aged 40–65 years	Weight loss ↓ Energy expenditure ↑ Fat oxidation ↑ Leptin ↓	[103]
Green tea extract	400 mg of green tea extract (491mg catechin containing 302 mg of EGCG) three times a day for 12 weeks.	To study the obesity-related hormonal peptides	>27 kg/m^2^	78 obese women aged between 16–60 years	Weight loss ↓ (non-significant compared to placebo) BMI ↓ (non-significant compared to placebo) Triglyceride ↓ LDL-cholesterol ↓ Adiponectin ↑ Ghrelin ↑	[104]
Green tea supplement along with endurance training	500 mg of green tea extract capsule (45% EGCG) once a day for 8 weeks.	Anti-inflammatory cytokines and adiponectin	>25 kg/m^2^	45 overweight men, aged 40–50 years	Bodyweight ↓ BMI ↓ Body fat percentage (BFP) ↓ Visceral fat area (VFA) ↓ IL-6 ↓ TNF-a (non-significant change) hs-CRP ↓ Adiponectin ↑	[105]
Decaffeinated green tea extract	530 mg of tea extract (containing 400 mg of catechin) twice daily for the following 6 weeks.	Ambulatory pressure and other metabolic markers	≥28 and ≤38 kg/m^2^	137 males, aged 49–60 years.	Body weight ↓ LDL ↓	[106]
Catechin- enriched green tea	609.3 mg catechin and 68.7 mg caffeine for 12 weeks	Effect on visceral fat	<24 to ≥40 kg/m^2^	118 subjects aged 20–65 years	Visceral fat ↓ Average visceral fat area ↓ Bodyweight ↓ Body fat ↓	[107]
Green tea EGCG	300 mg of EGCG for 12 weeks	Body composition related to obesity, markers for liver functional enzymes, and cardiometabolic risk factors	>30 to <40 kg/m^2^	38 obese premenopausal women	- Non-significant changes in all the factors in the treatment group compared to placebo in Bodyweight ↓ Energy ↓ Fat ↓ Total cholesterol ↓ LDL-cholesterol ↓	[108]
Decaffeinated green tea	Green tea extract (400 mg EGCG and a-lipoic acid) for 8 weeks	fat oxidation, cardio health, and body composition	25.0 to 29.9 kg/m^2^	27 obese subjects with regular physical activity	Fat oxidation ↑ Energy expenditure ↑ LDL-cholesterol ↓ Non-significant changes in cardio-metabolic indexes	[109]
Green tea	856.8 mg EGCG, 236.1 mg ECG, 115.5 mg for 6 weeks EGC)	Effect on low-density lipoprotein-cholesterol (LDL-c)	≥ 27 kg/m^2^	73 overweight women aged 18–65 years	LDL-cholesterol ↓ Leptin ↑ Non-significant changes in other markers related to obesity	[110]
Oolong tea	Oral uptake of 2 g of tea 4 times a day for 6 weeks	Effect on diet-induced obesity	N/A	102 obese subjects, aged 35–65 years	Bodyweight ↓ (70% > 1kg; 22% of subjects >3 kgs) Subcutaneous fat ↓ Plasma triglyceride ↓ Total cholesterol ↓	[75]
Green tea extract	EGCG at a dose of 150 mg twice a day for 8 weeks	EGCG on obesity, lipolysis, and browning of human white adipocytes	≥25 kg/m^2^	30 Thai obese subjects aged above 18 years	Plasma triglyceride levels ↓ No significant changes in- BMI, Bodyweight, Lipolysis, Uncoupling protein1 (UCP1), PPAR-γ agonist genes (hence no effect on browning effect)	[111]

## Data Availability

Not applicable.
